# COVID-19 Partners Platform—Accelerating Response by Coordinating Plans, Needs, and Contributions During Public Health Emergencies: COVID-19 Vaccines Use Case

**DOI:** 10.9745/GHSP-D-21-00460

**Published:** 2021-12-31

**Authors:** Angela K. Shen, M. Anne Yu, Ann Lindstrand, Sanjiv M. Baxi, Océane Jousset, Katherine O'Brien, Lucy Boulanger

**Affiliations:** a World Health Organization, Health Emergencies Programme, Geneva, Switzerland.; b Leonard Davis Institute of Health Economics, University of Pennsylvania, Philadelphia, PA, USA.; c World Health Organization, Department of Immunization, Vaccines and Biologicals, Geneva, Switzerland.; d University of California, San Francisco, San Francisco, CA, USA.; e McKinsey and Company, Geneva, Switzerland.

## Abstract

The World Health Organization COVID-19 Partners Platform represents the first step towards a new model of health crisis information sharing across stakeholders and could evolve into an engagement mechanism of choice for future cross-border public health emergencies.

## Key Messages

- The World Health Organization COVID-19 Partners Platform was conceived and implemented to support key operational engagement across health services and preparedness activities at the country, regional, and global levels.
- Use of the Platform is broadly applicable during public health emergencies but has proven as a critical asset to coordinate and accelerate deployment of vaccines for COVID-19 vaccination programs.
- The Platform is a dynamic environment created to accommodate the evolving needs of any pandemic response.

## INTRODUCTION

On January 30, 2020, the Director-General of the World Health Organization (WHO) declared the coronavirus disease (COVID-19) outbreak a public health emergency of international concern under the 2005 International Health Regulations.^1^ This triggered a cascade of events that led to the introduction of the COVID-19 Strategic Preparedness and Response Plans (SPRP) that emphasized the importance of responding to the crisis as “One UN.”^1^^–^^2^

While country readiness and response plans are key to tackling international public health emergencies, planning and coordinating across WHO member organizations have been challenging, and particularly so during an evolving global pandemic. To make the SPRP operational, WHO Health Emergencies Programme created the COVID-19 Partners Platform (referred to as the Platform; https://covid19partnersplatform.who.int/), conceived and implemented alongside other key operational support platforms and partnerships across health services and preparedness sectors (Table)^1^^–^^5^ to provide overall coordination and operational support to countries. This multidisciplinary effort aimed to guide the efforts of national and international partners, including donors, to support governments urgently to prepare, detect, and respond to epidemics and inform national planning while putting countries in the driver's seat.^6^ COVID-19 is a uniquely pressing emergency that can benefit from the real-time coordination of planning and tracking of activities that offers the following capabilities.

1. Consolidation of technical guidance and self-assessments enabling countries, territories, and areas to develop and refine their readiness and response plans in one place, avoiding siloes, difficult-to-access hard-copy storage, and email burden.
2. A secure web-based platform easily accessible even in low-bandwidth regions, with robust registered-user management processes under WHO standards for data privacy and protection.
3. Functionalities for all stakeholders across the public health landscape to share information and make decisions, including WHO Member States, United Nations agencies, implementation partners, suppliers, and donors.
4. Sustainability, as the Platform was conceived to be reproducible and scaled to prepare and respond to concurrent and future health emergencies.

**TABLE.** Key Operational Support Platforms and Partnerships

Coordination	COVID-19 Partners Platform: https://covid19partnersplatform.who.int/en/
Operational support and logistics and supply chains	World Health Organization COVID-19 Supply Chain System: https://www.who.int/emergencies/diseases/novel-coronavirus-2019/covid-19-operations
Training	World Health Organization's OpenWHO: https://openwho.org/
Surge health emergency workforce	Global Outbreak Alert and Response Network: https://extranet.who.int/goarn/
	World Health Organization Emergency Medical Teams Initiative: https://extranet.who.int/emt/
Public health intelligence	World Health Organization Epidemic Intelligence from Open Sources: https://www.who.int/initiatives/eios
Risk communication, community engagement, and infodemic management	World Health Organization Information Network for Epidemics (EPI-WIN) Updates: https://www.who.int/teams/risk-communication/epi-win-updates
	Global Outbreak Alert and Response Network COVID-19 Risk Communication and Community Engagement Collective Service: https://extranet.who.int/goarn/about-covid-19-rcce-collective-service
Research and innovation	World Health Organization R&D Blueprint: https://www.who.int/teams/blueprint/
	World Health Organization Access to COVID-19 Tools Accelerator: https://www.who.int/initiatives/act-accelerator

The Platform facilitates country planning aligned with global strategies, which has proved to be an important function for countries. The Platform enables the exchange of information, documentation of readiness in subcountry level areas, and ultimately, serves as a mechanism by which a broad set of donors can provide funding at a desired level of specificity as benchmarked by country needs. Most significantly, the Platform marked a step forward in shifting coordination of efforts from an “analog” to a “digital” response, enabling real-time access to information for countries' rapid planning and readiness, while also providing transparency into country plans, resource needs, and donor contributions. This was a first in global public health coordination.

The Platform enables the exchange of information, documentation of readiness in subcountry level areas, and serves as a mechanism by which a broad set of donors can provide funding at a desired level of specificity as benchmarked by country needs.

## THE PLATFORM

**FIGURE 1:**
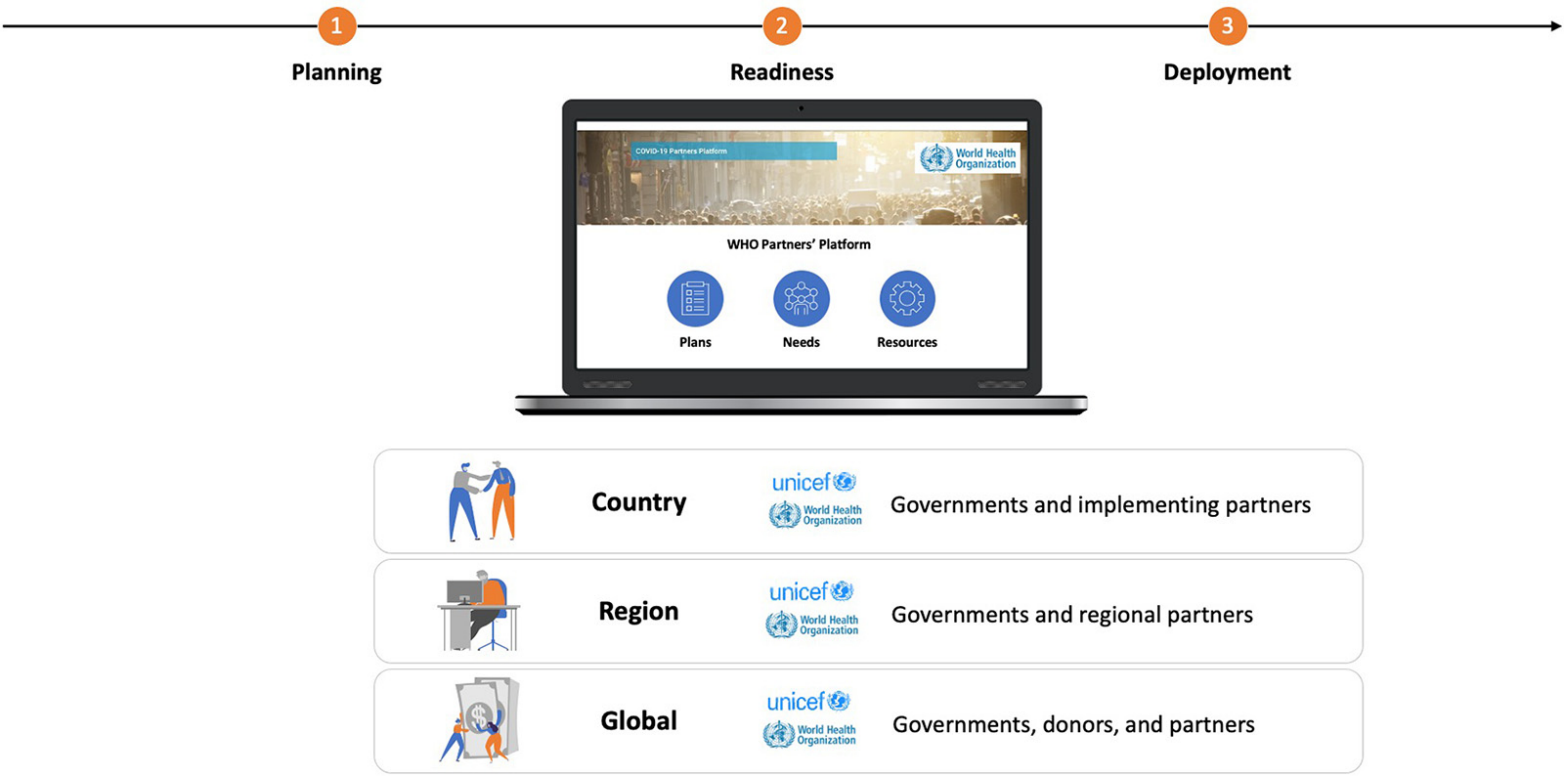
Supporting COVID-19 Vaccine Deployment Through Coordination on the WHO Partners' Platform Abbreviations: COVID-19; coronavirus disease; WHO, World Health Organization.

The Platform operates under 3 principles: transparency, collaboration, and efficiency.^4^^–^^6^ Transparency, in that all information is meant to be visible across users, meaning that the Platform aims to be “a common source of truth” for public health operational information on country readiness for and response to COVID-19. As a tool, it provides transparency for country resource needs and donor support through contributions. This transparency then facilitates collaboration among the donor community, technical community, and country by enabling direct connection among donors and recipients that then translates into internationally recommended actions supporting COVID-19 national response plans. By facilitating near real-time visibility on plans, needs, and resources, across national, regional, and global levels (Figure 1), the Platform supports the efficiency of operations and anchors the country at the center, enabling the “right people with the right information at the right time” to make decisions leading to timely and appropriate actions.

In parallel, early on in the pandemic response, the Platform facilitated requests for critical supplies by serving as a “doorway” to the WHO Supply Portal, an open marketplace based on standard packages of supplies (e.g., personal protective equipment kits and laboratory kits) that could be scaled up for the population at risk.^1^^–^^2^ The Platform provided a means by which countries could share not only their readiness and response plans but also their resource needs.^3^ This transparency on country resource needs provided visibility that could inform donors supporting global efforts. Up-to-date mapping of donor resources further allowed donors to focus on resource gaps in countries as they were identified.^4^^–^^6^

**FIGURE 2:**
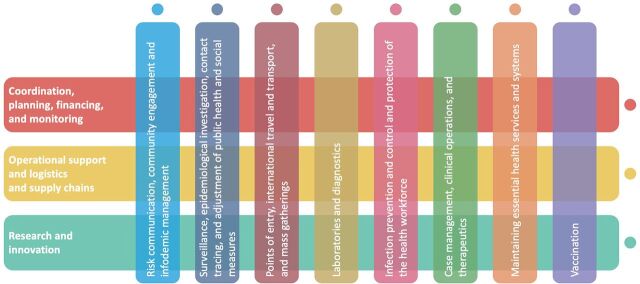
Eleven Key Pillars of the Strategic Preparedness and Response Plan 2021 Integrated Operations

The initial scope of the Platform was 8 key pillars targeted in the SPRP for country readiness in February 2020.^1^^,^^3^ These pillars encompassed well-established areas of public health readiness and response: (1) country-level coordination, planning, and monitoring; (2) risk communication and community engagement; (3) surveillance, rapid-response teams, and case investigation; (4) points of entry; (5) national laboratories; (6) infection prevention and control; (7) case management; and (8) operations support and logistics.^1^^–^^3^ As the pandemic escalated from April 2020 onward, a ninth pillar related to the continuation of essential health services was established as a paramount priority to address the increasing demand for care caused by COVID-19 while also maintaining essential health services (e.g., antenatal care).^3^^–^^6^ In February 2021, a critical tenth pillar, COVID-19 vaccine deployment, was added as a central strategy in the response effort.^4^^–^^6^ Underpinning these strategic pillars is also the recognition that “research and innovation,” as a de facto eleventh pillar, is a fundamental dimension of the response woven into the formal 10 pillars.^4^ Figure 2 illustrates these strategic pillars with interventions (vertical bars) and capacities (horizontal bars) that are facilitated by multidisciplinary national and subnational structures.^5^

The Platform is a web-based tool accessible by more than 4,000 active users representing 1,000 unique organizations spanning over 200 countries, territories, and areas. The platform is not a public application, but rather a tool reserved for public health responders. Of these countries, approximately two-thirds have shared their plans on the Platform, and 90 have shared their collective resource needs, which total more than US$9.3 billion across the first 9 pillars of health readiness and response. With this information, the Platform has become an information hub for global COVID-19 readiness and resource distribution where countries can interact transparently with global donors. This has resulted in more than US$9.1 billion in resources tracked to countries through the Platform from 77 donors.^4^ Without the Platform, such funding would normally be tracked through individual countries without a global matching of resources and needs and no systemwide visibility, thereby further contributing to what makes this platform a unique response tool.

The Platform has become an information hub for global COVID-19 readiness and resource distribution where countries can interact transparently with global donors.

What drives the distinct nature of the Platform is that countries are at the center, surrounded by key stakeholders including donors and implementing partners, enabling engagement with governments in a secure digital space through a clearinghouse of information central to decisions about response activities and funding to support response activities. All of this can then be tracked over time. Although a framework for evaluation and usability is currently under development, the application and use of the Platform in real-world settings is well illustrated in the current activity supporting COVID-19 vaccine deployment, through Pillars 10 and Pillar 11, the latest additions to the Platform.

## PILLAR 10: COVID-19 VACCINE DEPLOYMENT

As of June 17, 2021, Pillar 10 of the Platform was accessible by 1,184 users across 124 countries, territories, and areas. Of the total users of Pillar 10, approximately 59% are country administrators and 23% are implementing partners and donors. As the pandemic continues and the roll-out of COVID-19 vaccines extends inevitably into 2022 and 2023, it is expected that more users will come onto the Platform as interest heightens in supporting countries to meet global vaccination goals. The functions of the platform have served countries and the overall response effort by: (1) providing an overview of country needs based on their COVID-19 national deployment and vaccination plans (NDVPs), including resource needs; (2) serving as a mechanism to receive, review (when needed), and house strategic documents, as well as receive funding requests for operational support; and (3) mapping donor resource contributions to countries.

### 1. Provide an Overview of Country Needs Based on Their COVID-19 NDVP

While the race to develop vaccines has resulted in several safe and effective vaccines broadly available for use through emergency regulatory mechanisms, the last mile is to deploy these vaccines at a scale and pace that have never been attempted.^7^^–^^8^ This vaccination effort, the largest in history, will require intense coordination and collaboration globally, regionally, and at country levels. Toward this end, countries must also be prepared for vaccine deployment in unique ways as global supply constraints dictate a phased roll-out across priority populations of adults.^5^^,^^9^^,^^10^

Over time, with the rapid and accelerated development and production of COVID-19 vaccines, the Platform has taken on a coordinating role as part of COVAX, the worldwide initiative aimed at equitable access to the vaccines. COVAX is directed by Gavi, the Vaccine Alliance, the Coalition for Epidemic Preparedness Innovations, and WHO, with the United Nations Children's Fund (UNICEF) as a key delivery partner and the Pan American Health Organization as the procurement agent in the Americas. To support country delivery and readiness, the Platform provides country-specific information (e.g., total population, coverage, adsorption rate, and supply data) that is critical for country planning and management of doses and vaccination campaigns. These data support the fundamental principles of the Platform: to enable transparency, visibility, and action for countries, donors, and stakeholders alike.

To support country delivery and readiness, the Platform provides country-specific information that is critical for country planning and management of doses and vaccination campaigns.

**FIGURE 3:**
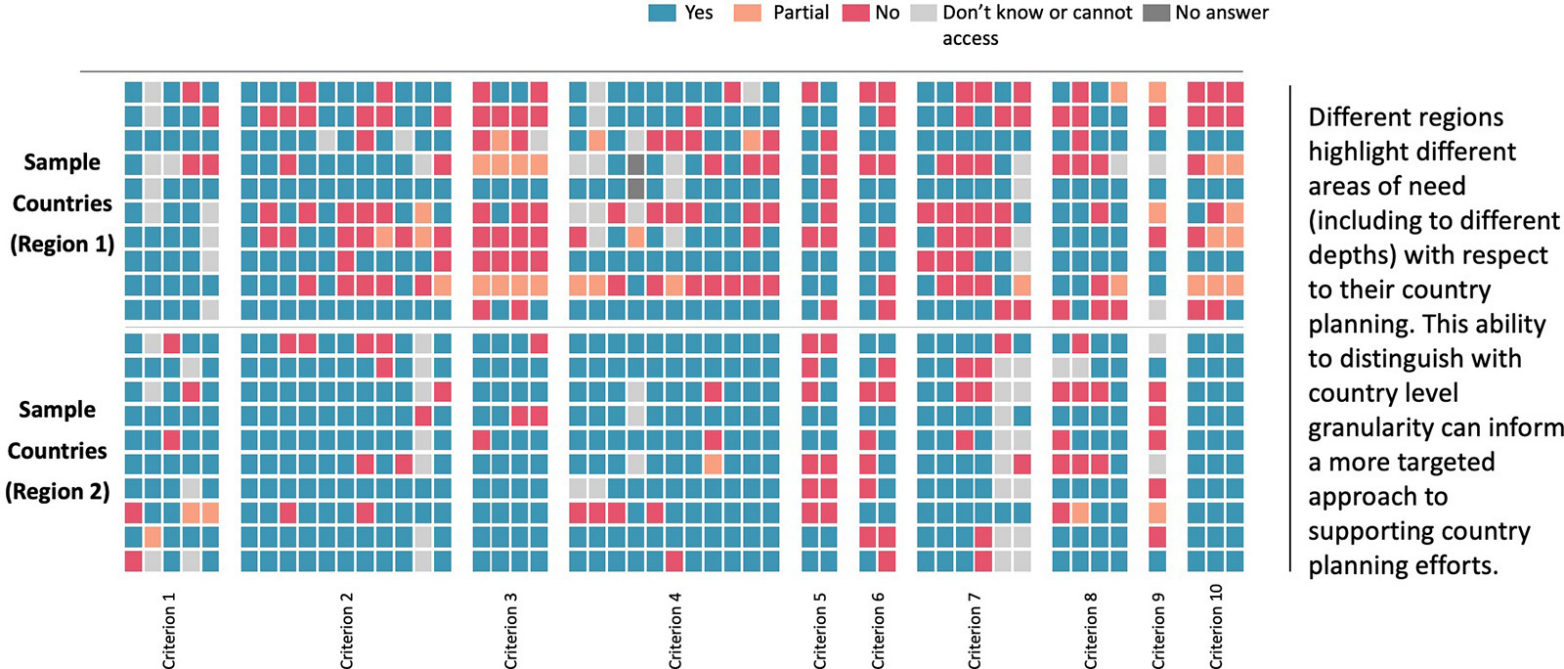
Heat Maps of National Deployment and Vaccination Plan Assessments With Blinded Samples of 10 Countries From 2 World Health Organization Regions

Through the leadership of WHO, UNICEF, and partners, 89 of 92 advance market commitment (AMC) countries, areas, and territories developed NDVPs, which serve as playbooks for organizing vaccination activities to strengthen immunization, health services, and health systems so that they can successfully introduce COVID-19 vaccination.^11^ Additional self-financing countries have also prepared NDVPs. The Platform became the single place for countries to upload an NDVP as a requirement to qualify for allocations of COVID-19 vaccines through COVAX. The Platform supported the rapid submission and review of 89 AMC and 19 non-AMC country plans. Review and approval of country readiness plans along with appropriate regulatory authorization are among the requisites for allocation and final release of vaccine doses from the COVAX facility to countries. Plans were uploaded onto the Platform within 3 days of the launch of the functionality, and initial rapid reviews of the NDVPs through regional review committees were completed within a week. The Platform allowed the experts of the regional review committees to review and assess the NDVPs— either directly online or offline—against predetermined criteria. This then allowed for analysis of areas of vulnerability in NDVPs, providing a global and regional snapshot of country readiness that could highlight areas of need for technical assistance and further support. A heat map of these assessments, stratified by WHO region (Figure 3), illustrates the ability to do this type of analysis.

While regional review committees were set up to execute this review function in initial NDVP submissions, additional revisions to the NDVP have not been reviewed, though the Platform can easily accommodate this function. Further, the Platform has the capacity and capability to receive as many updated plans as countries wish to submit, particularly as the evolving needs of the pandemic may dictate updates and revisions. Revisions may include changes to national targets and milestones and expansion of target groups including hard-to-reach, underserved, refugees, and migrants; lessons learned from initial roll-out including coordination management of multiple products; human resource surge requirements; and expansion and adaptations to cold chain and logistics or any other dimension of plans a country may wish to revise.^10^

Notably, 81 NDVPs were also reviewed by WHO humanitarian experts supporting fragile, vulnerable, and conflict countries to determine if there was adequate inclusion of vulnerable populations (e.g., refugees, asylum seekers, and those dedicated to relieving their suffering). Because of the ability to access country plans and provide technical support to countries for this area of planning, revisions to country plans were made immediately. Weeks later, Gavi approved 5% of COVAX AMC funding for doses to be deployed through COVAX via a “humanitarian buffer.” This mechanism seeks to address vulnerable populations that might not be included in other allocation and access mechanisms (e.g., in instances of state failure and conflict and areas controlled by non-state armed groups and thus inaccessible to governments).

Country NDVPs also serve as guide rails for the costing of activities needed to resource programs adequately to vaccinate their populations successfully. Then, donors can understand the costs for each activity and how to deploy new and strengthened existing systems around these costing components. Countries can then assess and align within available domestic resources around their plan without crowding out other existing services all the meanwhile minimizing duplication. The use of bottom-up costing tools that fit around various modalities (e.g., fixed facility and campaigns), as well as population priority groups, is key to ensuring campaigns are not underresourced.

To fully understand the resource needs for implementing a country's NDVP, countries costed their activities. Countries are encouraged to use the WHO COVID-19 vaccine introduction-and-deployment costing tool (CVIC tool 2.2, (https://www.who.int/publications/i/item/10665337553) and upload their needs onto the Platform.^12^ Countries could also choose to provide outputs manually through drop-down menus across 9 common costing categories using whatever method or tools they wished. The process of developing consensus around these 9 common costing categories galvanized partners and donors to agree on a unified and standardized way for the Platform to display countries' needs and donor contributions. No successful campaign can occur without a vaccine, but a vaccination program is much wider than the product alone. Notwithstanding the tremendous global effort to ensure equitable distribution of vaccines, the highest risk to the program is not having enough resources to implement the vaccination program successfully.

### 2. Serve as a Mechanism for Receiving, Reviewing, and Storing Strategic Documents and Receiving Funding Applications

**FIGURE 4:**
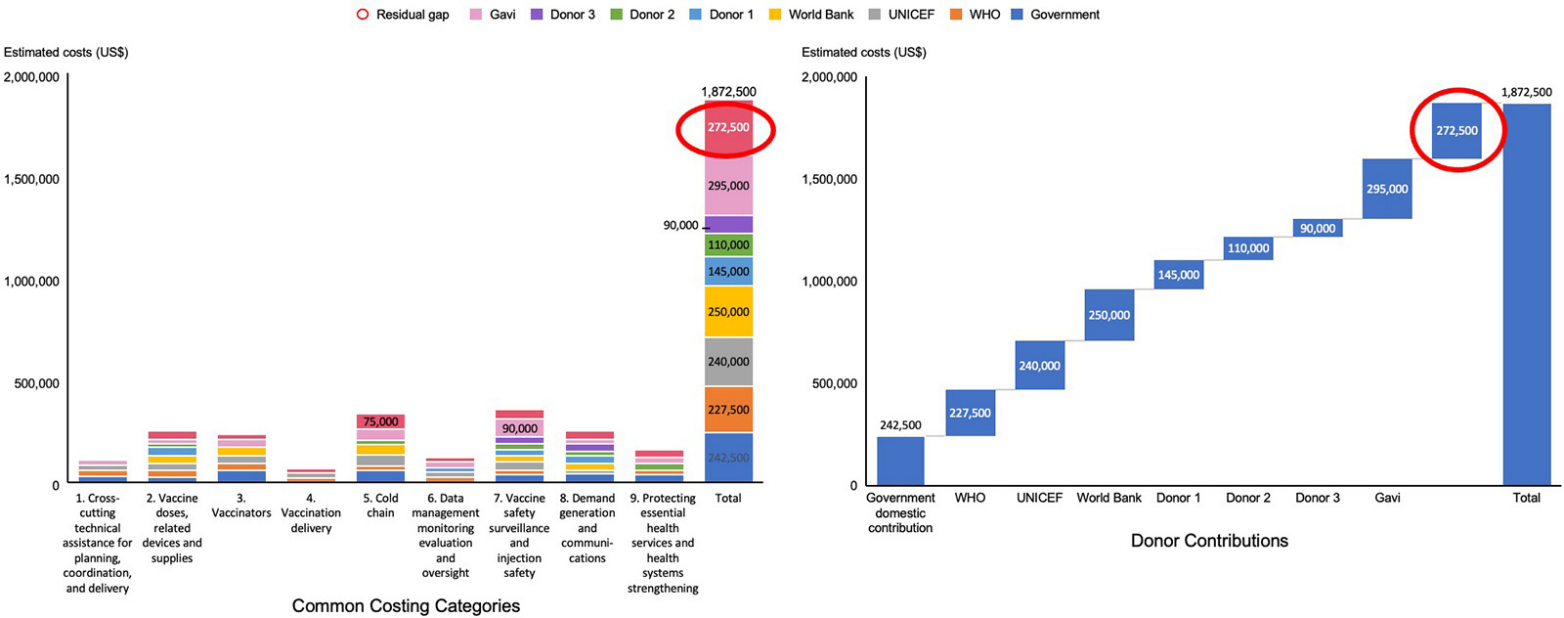
Illustrative Example of Country X Estimated Costed Needs and Global Contributions

The first function provides a country view and context of needs and resources important to country planners and funders who wish to support countries. The Platform has been successfully used as a way for countries to apply for Gavi and UNICEF COVID-19 delivery support for the deployment of vaccines. The data that are captured as part of the application form asks countries to explain their needs, relative to their NDVPs. It also includes the total costed needs for their COVID-19 vaccination program as well as domestic and external sources of revenue. In doing so, there is a quantified residual gap of financing that is displayed of needs yet to be met. This residual gap is shown on a dashboard by the Platform, stratified by costing categories and by domestic government and donor contributions (Figure 4) to visualize the management of funding support and further amplify the principle of transparency. Figure 4 demonstrates how the Platform has evolved to meet the needs of donor partners and countries alike, beyond getting a plan in place.

### 3. Map Donor Contributions

The impact of COVID-19 on vulnerable countries has been devastating, threatening gains in economic growth and development while at the same time exacerbating existing inequities, particularly in the world's poorest economies. Donors across the immunization and development community have committed to addressing the goals of equitable access to vaccines through both broad-scale vaccination programs and resilient recovery (e.g., reducing extreme poverty).^13^^–^^16^ The ability for donors to have visibility across other donor contributions provides a means to address gaps in need across the immunization program. The Platform provides this visibility across 9 major common domains of need for immunization programs: (1) technical assistance for planning, coordination, and delivery; (2) vaccine doses and related devices and supplies; (3) human resources including training; (4) vaccination delivery; (5) supply chain and cold chain; (6) data management, monitoring and evaluation, and oversight; (7) vaccine safety surveillance and injection safety; (8) demand generation and communication; and (9) additional interventions such as maintaining essential health services.^4^ All donors (e.g., development banks and philanthropic foundations), regardless of the type of support (e.g., bilateral or multilateral), and all users of the Platform can see these mapped donor contributions.

The ability for donors to have visibility across other donor contributions provides a means to address gaps in need across the immunization program.

As the global donor community ramps up support for COVID-19 vaccine deployment in countries,^11^ it is critical to see the granular distinctions of which needs are resourced. Otherwise, it will become difficult to ensure the total needs of countries are met. For example, some donors may wish to finance the purchase of COVID-19 vaccines, which represent the largest proportion of costs that need to be financed, in addition to supporting widespread testing, improved treatment, and strong health systems critical to saving lives and supporting global economic recovery. Meanwhile, other donors may wish to support technical assistance or delivery and operational costs. The donor resource mapping on the Platform already provides significant insight into this global view.

## LESSONS LEARNED

The Platform represents a real improvement from previous platforms and tools, but it is only the first step. Care will need to be taken to learn lessons from its implementation and to make refinements appropriately. Some critical lessons are already apparent, particularly that the Platform can rapidly respond to the needs of users through the dynamic response (i.e., serve as a platform to apply for funding support). In terms of further refinement of the platform, the value of diverse perspectives is clear: including a full spectrum of stakeholder input to support the broad needs of diverse groups coming together around a single challenge. Human-centered design thinking, although not new, has proven its particular importance during an emergency—designers make all the difference in the experience. The design and launch of the Platform are only the beginning: user adoption requires time (and patience), training, communication, and support (e.g., aid to translate recommendations to country context, 1-on-1 tailored sessions, and translations). There will always be a continuing need to adjust to the pandemic in real-time to make sure that the platform stays relevant and continue to be used widely. High adoption rates are critical for the Platform to deliver on its full potential. In addition, continued attention needs to be given to the safety and governance of the platform.

In terms of preparation for the next pandemic, there is a clear need to maintain a communication and engagement platform in readiness. Data and metrics that allow countries to assess their readiness to face pandemics must be regularly generated, collected, and analyzed, even during “peacetime.” The Platform is a good candidate for this role if it continues to evolve as is required by public health emergency response.

## Notes

**Peer Reviewed**
